# Mortality and Recovery of Renal Function in Acute Kidney Injury Patients Treated with Prolonged Intermittent Hemodialysis Sessions Lasting 10 versus 6 Hours: Results of a Randomized Clinical Trial

**DOI:** 10.1155/2018/4097864

**Published:** 2018-08-13

**Authors:** Bianca Ballarin Albino, Mariele Gobo-Oliveira, André Luís Balbi, Daniela Ponce

**Affiliations:** ^1^Botucatu School of Medicine, University of São Paulo State (UNESP), Brazil; ^2^Course of Medicine, University of São Paulo (USP), Bauru, Brazil

## Abstract

**Purpose:**

This trial aimed to compare mortality and recovery of renal function in acute kidney injury (AKI) patients treated with different durations of prolonged hemodialysis (PHD) sessions (6 h versus 10 h).

**Methodology:**

We included patients with sepsis-associated AKI, >18 years, who are in use of a norepinephrine (lower than 0.7 ucg/kg/min).

**Results:**

One hundred and ninety-four patients were treated with 531 sessions of PHD (G1=104 and G2=90 patients). The two groups were similar in age and SOFA. There was no significant difference in hypotension, hypokalemia, and anticoagulation during PHD sessions. The two groups showed differences in filter clotting, hypophosphatemia, and treatment discontinuation (12.3 versus 23.1%, p=0.002; 15.5 versus 25.8%, p=0.005; and 7.9 versus 15.6%, p=0.008, respectively). There was no difference in fluid balance (FB) before and after PHD sessions. Death and complete recovery of renal function were similar (81.3 versus 82.2%, p=0.87 and 21 versus 31.2%, p=0.7, respectively). At logistic regression, the positive FB before and after dialysis was identified as risk factor for death, while volume overload after three PHD sessions and predialysis creatinine were negatively associated with recovery of renal function in 28 days.

**Conclusion:**

There was no difference in the mortality and recovery of renal function of AKI patients submitted to different durations of PHD and sessions lasting 10 h presented higher filter clotting, hypophosphatemia, and treatment discontinuation. ISRCTN Registry number is ISRCTN33774458.

## 1. Introduction

Acute kidney injury (AKI) is a complex and frequent syndrome in septic patients admitted to intensive care units (34%). Despite the reduction of its mortality rate in recent years, it still remains high, reaching 62% in critical patients and 80% in those who require dialysis [1–5].

Peritoneal dialysis (PD) and hemodialysis (HD) are options for acute kidney injury support. Depending on their duration and flow of blood and dialysate they can be classified as conventional intermittent hemodialysis (IHD), prolonged intermittent hemodialysis (PHD), and continuous renal replacement therapy (CRRT) [6, 7]. So far there has been no evidence of one method being superior to the others.

PHD consists of a hybrid method with IHD characteristics, such as the use of machines and filters similar to those used in the treatment of chronic dialysis patients. It also has CRRT characteristics, such as smaller flow of blood and dialysate, between 70-250 ml/min and 70-300 ml/min, respectively [8–10]. The duration of PHD therapy varies between 6 and 18 hours [7, 9–11]. This method is considered to be as effective as conventional IHD and CRRT in regard to metabolic and volume control. It also has advantages when compared to CRRT, such as a lower cost, a reduced need for anticoagulation, and time optimization, with the possibility of having the patient undergo exams and procedures away from the ICU [12–14].

At the moment the literature does not include any studies that have evaluated and compared mortality rates and recovery of renal function in patients with AKI treated with PHD sessions of different durations.

This clinical trial was designed to evaluate and compare the recovery of renal function, mortality, and intra- and postdialysis complications in critically ill AKI patients undergoing PHD sessions lasting 6 or 10 h. We hypothesized that PHD sessions lasting 10 hours would cause less hypotension than PHD sessions lasting 6 hours, leading to faster partial recovery of renal function.

## 2. Methods

### 2.1. Studied Population

This is a randomized clinical trial conducted from January 2012 to March 2016 in patients enrolled in the Brazilian University Hospital. The protocol was approved by the Institutional Ethical Committee (CAAE Protocol: 28146714.6.0000.5411). Written informed consent was obtained from patients or their next of kin. The study also was registered in the ISRCTN Registry under the number ISRCTN33774458.

Patients were eligible for enrolment if they were 18 years of age or older, with AKI associated with sepsis and on a noradrenaline dose lower than 0.7 *μ*g/kg/min. AKI was defined according to of KDIGO [15]. Exclusion criteria were chronic kidney disease stages 4 and 5, previous chronic dialysis, kidney transplantation, patients previously treated with any acute dialysis during ICU stay, pregnancy, and taking noradrenaline dose higher than 0.7 *μ*g/kg/min. These last patients were excluded because they could not tolerate actual ultrafiltration (UF) of 300–500 mL/h and because of that, they were treated with CRRT.

### 2.2. Criteria for Initiating and Stopping PHD

The indications for dialysis were uraemic symptoms, BUN level > 100 mg/dL (azotaemia), volume overload, electrolyte imbalance (potassium > 6 mEq/L after clinical treatment), or acid-base refractory disturbances (bicarbonate < 10 mEq/L after reposition).

A patient was considered for enrolment if the judgment of the treating nephrologists was that he or she required dialysis and the mean arterial blood pressure (BP) was higher than 80 mmHg, with a noradrenaline dose lower than 0.7 *μ*g/kg/min in the 8 hours preceding randomization.

The patients were randomly assigned to two groups, according to the treatment duration prescribed. The randomization was computerized by the randomization.com website.

Group 1: Patients treated with PHD sessions lasting 6 hours

Group 2: Patients treated with PHD sessions lasting 10 hours

The hemodialysis sessions were accompanied by the same research team until the patient's final clinical outcome (recovery of renal function, change of dialysis method, or death).

After diagnosing the sepsis-associated AKI and indicating PHD for dialysis, the central venous catheter was implanted to initiate treatment. The PHD session lasted 6 or 10 hours according to randomization and, for practical reasons, it was decided that PHD would be carried out 6 days a week (Monday–Saturday).

We used proportion machines (Fresenius 4008) and capillary polysulfone membranes (surface areas of 1.0 and 1.2 m^2^) for the sessions.

The PHD sessions were performed with blood and dialysate flows of 200 and 300 mL/min, respectively. For Group 1 we used FX 80 capillary dialyzers, while for Group 2 we used FX 60 capillary dialyzers in order to minimize the difference between the dialysis doses provided. During the sessions, the patients were anticoagulated with a 50 to 100 IU/kg bolus dose of heparin, and then with 500 to 1000 IU/hour in the following hours. In cases of contraindication to anticoagulation, the system was washed with 50 ml of 0.9% sodium chloride every 30 minutes, throughout the entire procedure. The concentrations of bicarbonate (26-36 mEq/L), potassium (1-3 mEq/L), sodium (140-145 mEq/L), and calcium (2.5 to 3.5 mEq/L) of the dialysis bath were adjusted according to the exams and individual needs of the patients. The ultrafiltration rate (UF) did not exceed 500 ml/hour and the bath temperature ranged from 35 to 35.5°C.

We evaluated hypotension and filter clotting as intradialytic complications. Hypotension was defined as systolic blood pressure (BP) below 90 mmHg, or as a sudden BP drop of 20 mmHg. Filter clotting was defined as the presence of blood clots in the circuit, composed of a dialyzer and lines, precluding the continuation of therapy. As preventive measures to hypotension we used UF rates not exceeding 500 ml/h, dialysate temperature between 35 and 35.5°C, and high dialysate sodium concentration (140-145 mEq/L). When we observed the presence of thrombi and blood clots in the system, we conducted saline flushes or administered an extra dose of heparin to prevent coagulation, according to medical prescription. As postdialysis complications we evaluated the presence of hypokalemia and hypophosphatemia, characterized by serum levels below 3.5 mEq/l and 3.5 mg/dl, respectively.

We evaluated the outcomes of death and recovery of renal function in 28 days. The recovery of renal function was assessed using the relationship between the reference creatinine level and the creatinine level at 28 days after hospital discharge (reference Cr/Cr at 28 days) and classified the recovery as complete if above 0.9, partial if between 0.5 and 0.9, and as nonrecovery if below 0.5. [16]

We collected the following clinical data: name, gender, age, race, presence of comorbidities (diabetes, chronic kidney disease, and hypertension), primary diagnosis, sepsis etiology, and AKI specific prognostic score (ATN-ISS) [17], Sequential Organ Failure Assessment (SOFA) [18], date of hospitalization, PHD starting date, concentration of vasoactive drug before and after PHD. We quantified urea, creatinine, sodium, potassium, phosphorus, calcium, and venous gas every day, before and after HD. Posttreatment BUN levels were measured by the slow flow method (with blood pump speed reduced to 50 mL/min). Blood samples were obtained from the arterial sampling port before the blood reached the dialyzer. HD adequacy was determined by using urea kinetic modelling based on Kt/V [19]. The delivered dose was determined by the single-pool Kt/V value, corrected for actual UF but not for the reappearance of urea nitrogen [19].

As dialytic therapy data we evaluated the number of sessions performed and of filters used, blood and dialysate flows, volume of UF prescribed and obtained, urea removal rate, BP every 30 minutes, and the presence of hypotension and filter clotting as previously defined. To solve hypotension during PHD, we applied protocols which included saline infusion, decreasing or discontinuing UF and increasing the vasoactive drug, according to the clinical and volume conditions of the patient. Therapy was interrupted when, despite taking these measures, hemodynamic instability persisted and presented risks to the patient.

Dialysis was interrupted when there was partial recovery of renal function (dialysis-independent) defined as restoration of urine output higher than 1000 mL/24 h associated with a progressive fall in serum values for creatinine (<4 mg/100 mL) and BUN (<50 mg/dL), a need to change dialysis method because of infectious, mechanical, or haemodynamic complications, more than 30 days of follow-up, or death.

### 2.3. Statistical Analysis

Considering alpha error as 5%, beta error as 20%, statistical power of the test as 80%, and detecting a 15% mortality rate difference between groups, the sample size for each group was calculated as 94 patients.

We described the variables with normal distribution as mean value ± standard deviation and the variables with nonnormal distribution as mean value and interquartile range.

We performed comparisons of the continuous variables between the two groups using Student's t-test for data with normal distribution and the Mann–Whitney test for nonnormal data. For the comparative analysis of categorical variables we used the Chi-squared tests. For comparing variables by session between the groups we used the mixed model of repeated measures over time with an adjustment for Tukey's test. We used univariate and multivariate linear regression for the association of risk factors for death and nonrecovery of renal function in 28 days.

We considered a 5% significance level in all of the tests performed.

For data analysis we used the SAS program for Windows, version 9.2 (developed in 2009, in Cary, North Carolina, USA).

## 3. Results

A hundred and ninety-four patients with sepsis-associated AKI received 531 PHD sessions. The mean age was 60.8 ± 14.9 years, and 69.5% of the patients were male. The main infection focus was pulmonary (41.2%) and hypertension was the most prevalent of the comorbidities (52.5%). The specific prognosis index for AKI, the ATN-ISS, was 0.77 ± 0.1 and the mean SOFA score was 14.2 ± 2.9.

G1 was composed of 104 patients, treated for 276 sessions. G2 was composed of 90 patients, treated for 255 sessions. When comparing clinical characteristics, both groups were similar in relation to the predominance of males (70.1% in G1 and 68.8% in G2, p=0.84), mean age of 61.4 ± 14.4 years in G1 and 60.5 ± 15.5 years in G2 (p=0.55), presence of comorbidities such as hypertension and diabetes (55.7% versus 48.8%, p=0.33 and 27.8% versus 26.6%, p=0.84, respectively), specific prognosis for ATN (ATN-ISS), and SOFA (0.76 ± 0.1 versus 0.77 ± 0.2, p=0.87 and 14.1 ± 3 versus 14.4 ± 2.9 p=0.47, respectively). In Group 1, pulmonary infection focus was prevalent (41.3%), while in Group 2, the main focus was abdominal (42.2%, p = 0.07). Most patients were on mechanical ventilation, 94.2% in Group 1, compared to 93.3% in Group 2 (p = 0.96). The groups were similar regarding initial and final doses of vasoactive drug (0.56 versus 0.55, p = 0.97 and 0.69 versus 0.7, p = 0.91, respectively) as shown in Table 1.

Table 2 shows the presence of dialysis complications during hemodialysis sessions, in general and divided by groups. The main complication among them was hypotension (50%), followed by filter clotting, hypophosphatemia, and hypokalemia, which happened in 17.5%, 20.5%, and 11.2% of the sessions, respectively. There was a significant difference between the groups in relation to filter clotting (12.3 versus 23.1%, p = 0.002), hypophosphatemia (15.5 versus 25.8%, p = 0.005), and hemodialysis session interruption due to the presence of persistent hypotension after refractory measures (7.9 versus 15.6%, p = 0.008). Both groups were similar in relation to hypotension (46.7 versus 53.7%, p = 0.13), hypokalemia (11.5 versus 10.9%, p = 0.93), and the use of anticoagulation in the PHD sessions (45.2 versus 36.8%, p = 0.06).

The main outcome was death, present in 81.7% of the general population. Recovery of renal function (RF) was assessed among the survivors: 25.7% presented complete recovery, 68.5% presented partial recovery, and 5.7% presented nonrecovery. There was no difference between the groups in relation to outcomes (death: 81.3 versus 82.2%, p = 0.87; complete recovery of FR: 21 versus 31.2%, p = 0.7; partial recovery of RF: 68.4 versus 68.7%, p = 0.7; nonrecovery of RF: 10.5 versus 0%, p = 0.48), as shown in Table 3.

The metabolic and volume controls of the AKI patients treated with PHD sessions lasting 6 and 10 hours were assessed after the first three sessions and are presented in Table 4. The target metabolic and volume control was reached in both groups we studied with urea lower than 120 mg/dl, pH above 7.3, minimum weekly Kt/v of 3.9, and cumulative fluid balance close to zero after 3 sessions. The groups showed differences in the values of urea reduction ratio (URR) on the first and third sessions (S1: 0.6 ± 0.1 versus 0.68 ± 0.1; p < 0.001, S3: 0.56 ± 0.1 versus 0.62 ± 0.1; p = 0.03), being higher in G2. Potassium and phosphorous serum values were (4.3 ± 0.8 versus 3.9 ± 0.6, p = 0.04; 5.8 ± 2.2 versus 4.1 ± 1.6, p = 0.009, respectively), being lower in G2. The rate of ultrafiltration (UF) prescribed in the first three sessions was (S1: 2064 ± 927 versus 2580 ± 1000, p= 0.0002; S2: 2262 ± 852 versus 2626 ± 1123, p=0.02, and S3: 2217 ± 755 versus 2656 ± 1004, p=0.03) and real UF in the first session was (1791±963 versus 2345 ± 1017, p = 0.0006), which were higher in G2. However, the UF in ml/h was lower in G2 in all sessions we studied. The other parameters assessed were similar between the groups. Fluid balance was assessed before the first and third sessions of PHD, and there was no difference between the groups (S1: 3.27 ± 1.9 versus 3.47 ± 1.7, p = 0.47 and S3: 1.33 ± 2.6 versus 0.47 ± 2.4, p = 0.09).

A logistic regression was conducted for the death outcomes and the variables of weight, SOFA, fluid balance before and after 3 sessions, presession potassium, and the presence of hypokalemia were identified as risk factors. After the multivariate analysis, the association remained only for fluid balance before and after 3 sessions. This data is included in Tables 5 and 6.

Similarly, the logistic regression was carried out for the recovery of renal function in 28 days, and the values of fluid balance before and after 3 sessions and creatinine presession presented significant negative association, which remained after the multivariate analysis. This data is shown in Tables 7 and 8.

## 4. Discussion

This clinical trial study aimed to assess and compare the mortality rate and recovery of renal function in critical patients with AKI, treated with PHD sessions of different durations (6 and 10 hours). There are very few studies on PHD in the literature and, until now, none of them compared the clinical evolution of patients in PHD of different durations.

Hypotension was the most common complication and occurred in 50% of the sessions, despite the use of precautionary measures, such as low dialysate temperature, high sodium concentrations, and UF rate not exceeding 500 ml/h. Similar results were reported by Fieghen et al. [20], Ponce et al. [21], And Albino et al. [22].

However, there was no difference between the groups treated in our study. The longest session duration time in Group 2 resulted in a lower ultrafiltration rate (ml/hour), though it did not prevent and/or improve the frequency of hypotension. The dose of the vasoactive substance was higher at the end of the PHD sessions, when compared to the dose at the beginning, in an attempt to keep arterial pressure steady during therapy, and it was similar between the groups.

The second most frequent dialysis complication in our study was filter clotting, which occurred in 11.2% sessions, similar to the data reported in the literature [3, 12, 21, 22]. The use of anticoagulation in the PHD sessions was carried out according to the comorbidities and bleeding risk related to the patient. Anticoagulation occurred in 41.2% of the sessions and there was no difference between the groups.

The prevalence of filter clotting was different between the groups as was treatment discontinuation. These facts may be related to the longer duration of treatment of Group 2 patients, making them more susceptible to the persistence of intradialytic complications.

Hypokalemia and hypophosphatemia are postdialysis complications that are rarely addressed in the previous studies conducted on PHD, which complicates the analysis and comparison of the results obtained in our study. Marshall et al. [23] analyzed 145 PHD sessions in 37 patients and found hypokalemia and hypophosphatemia in 7 (4.8%) and 18 (12.4%) occurrences, respectively, and these results are similar to those found in our study. Similarly, Palevsky et al. [24] found a prevalence of hypophosphatemia in 12.4% of the patients treated with PHD in their ATN study.

There was a difference between the groups we studied in relation to hypophosphatemia, probably associated with the longer therapy duration and consequently greater removal of solutes, and the groups were similar in relation to hypokalemia.

The target metabolic and volume control was reached in both groups we studied. However, there was difference between the groups regarding the URR and Kt/V, which were higher in group 2. Despite the use of capillaries with a smaller area for Group 2, due to the 10-hour treatment duration, these patients received a slightly higher Kt/v.

Although the Kt/V was higher in Group 2, the mortality rate was similar between the groups, in accordance with previous studies that showed that more intensive dialysis is not associated with better outcomes [25, 26].

The ultrafiltration value was prescribed according to the fluid balance of the patient, and it ranged between 1500 and 3000 ml per session. There was a difference between the groups. Group 2 had higher prescribed and actual UF. However, the fluid balance was similar between the studied populations after 3 sessions of therapy. There was no difference in cumulative fluid balance before and after 3 dialysis sessions between the groups.

Death occurred in 81.7% of the general population and there was no difference between the groups. The mortality rate we found was higher than those reported in previous studies carried out in European countries and in North America, such as in a study conducted in Toronto by Kitchlu et al., who observed death in 54% of patients treated with PHD [12]. Our mortality rate, however, was similar to those observed in critical patients with AKI in developing countries. In Brazilian studies, mortality of AKI patients that underwent dialysis ranged from 67 to 85% [3, 22, 27, 28]. This data is similar to that described by George et al., who performed a study in India and obtained mortality over 75% in patients with AKI [29].

Considering we included patients with sepsis-associated AKI and elevated prognostic indexes (ATN-ISS and SOFA of 0.77 ± 0.1 and 14.2 ± 2.9, respectively), the patients studied were in severe conditions, which justifies the unfavorable outcomes. It is important to emphasize that we only evaluated septic patients, which was not made in other studies.

We identified cumulative fluid balance before dialysis and after 3 sessions of PHD as the only death-associated factor. These results are in agreement with previous studies that reported low urine output, fluid overload, and sepsis are associated with worse prognostic of AKI patients [21, 26, 30, 31]. Clinical data show that positive fluid balance and oliguria can contribute negatively to prognosis lung, leading to increased time of invasive mechanical ventilation, durations of hospitalization, and mortality [30, 31].

In our study, we could not identify dialysis dose as a risk factor for death, in agreement with Palevsky et al. [25] and Bellomo et al. [26] in the trials ATN and RENAL, respectively.

Recovery of renal function was assessed among the survivors: 25.7% had complete recovery and 68.5% had partial recovery. Similar results reported that a quarter of the patients obtained complete recovery of renal function after 30 days. In our study, the predialysis creatinine value and fluid balance after 3 sessions were identified as risk factors for the recovery of renal function. Hamzić-Mehmedbašić et al. identified that the female gender, comorbidities, and sepsis were risk factors for a worse evolution of renal function [32].

Our study presents several limitations, such as the small number of studied patients and its execution in a single center. Due to the different duration of treatment between the groups, we were not able to perform the randomization blindly. The assessment of long-term survival was also not performed. Despite these limitations, this was the first study to assess the clinical evolution of patients with AKI treated with different durations of PHD [33].

In conclusion, our results show that mortality and recovery of renal function are similar between the groups treated with PHD lasting 6 and 10 h. However, Group 2 showed higher incidence of dialysis complications, such as filter clotting and hypophosphatemia, probably related to the extended duration of therapy. Therefore, there is no benefit in treating patients with 10-hour sessions.

Future work in this area should aim to clarify factors that inform decision-making around time of PHD modality. Larger and trial studies will need to clarify the impact of PHD on patient survival and recovery of renal function.

## Figures and Tables

**Table 1:** Clinical and laboratory characteristics of patients with AKI treated with PHD.

**Parameters**	**General (n = 194)**	**G1 (n = 104)**	**G2 (n = 90)**	**p value**
Age (in years)	60.8 ± 14.9	61.4 ± 14.4	60.2 ± 15.5	0.55
Males, n (%)	135 (69.5)	73 (70.1)	62 (68.8)	0.84
Weight	74.8 ± 22.5	74.1 ± 24	75.6 ± 20.8	0.63
**Infectious focus n (%)**				
Pulmonary	80 (41.2)	43 (41.3)	37 (41.2)	0.97
Abdominal	69 (35.5)	31 (29.8)	38 (42.2)	0.07
**Comorbidities, n (%)**				
(i) SAH	102 (52.5)	58 (55.7)	44 (48.8)	0.33
(ii) DM	53 (27.2)	29 (27.8)	24 (26.6)	0.84
(iii) CKD	19 (9.7)	12 (11.5)	7 (7.7)	0.37
ATN-ISS	0.77 ± 0.1	0.76 ± 0.1	0.77 ± 0.2	0.87
SOFA	14.2 ± 2.9	14.1 ± 3	14,4 ± 2,9	0.47
Pre-dialysis FB (l)	3.36 ± 1.8	3.27 ± 1.9	3.47 ± 1.7	0.47
Ur (mg/dl)	155.3 ± 105.7	158.6 ± 59.5	151.6 ± 141.2	0.64
Cr (mg/dl)	3.7 ± 1.5	3.7 ± 1.4	3.6 ± 1.7	0.64
K (mEq/L)	4.7 ± 1	4.7 ± 1	4.8 ± 1	0.48
Bic (mEq/L)	19.1 ± 4.6	18.8 ± 4.7	19.5 ± 4.6	0.39
Mechanical Ventilation	182 (93.8)	98 (94.2)	84 (93.3)	0.96
Initial vasoactive drug dose	0.55 ± 0.18	0.55 ± 0.17	0.56 ± 0.19	0.97
Final vasoactive drug dose	0.69 ± 0.19	0.69 ± 0.16	0.70 ± 0.20	0.91

Values are presented in frequency, mean values and standard deviation, median, and proportions.

AKI: acute renal injury, PHD: prolonged hemodialysis, SAH: systemic arterial hypertension, DM: diabetes mellitus, CKD: chronic kidney disease, ATN-ISS: acute tubular necrosis individual severity score, SOFA: sequential organ failure assessment score, FB: fluid balance, Ur: urea, Cr: creatinine, K: potassium, Bic: bicarbonate, and UF: ultrafiltration.

**Table 2:** Dialysis complications by PHD sessions according to the groups studied.

**Complications**	**General** **(n = 531)**	**G1** **(n = 276)**	**G2** **(n = 255)**	**p value**
Hypotension, n (%)	266 (50)	129 (46.7)	137 (53.7)	0.13
Filter clotting, n (%)	93 (17.5)	34 (12.3)	59 (23.1)	0.002
Hypokalemia, n (%)	60 (11.2)	32 (11.5)	28 (10.9)	0.93
Hypophosphataemia, n (%)	109 (20.5)	43 (15.5)	66 (25.8)	0.005
Use of anticoagulation, n (%)	219 (41.2)	125 (45.2)	94 (36.8)	0.06
Treatment discontinuation, n (%)	62 (11.6)	22 (7.9)	40 (15.6)	0.008

Values are presented in proportions.

PHD: prolonged hemodialysis.

**Table 3:** Main outcomes of patients with AKI treated with PHD according to the duration of the PHD sessions.

**Outcomes**	**General** **(n = 194)**	**G1** **(n = 104)**	**G2** **(n = 90)**	**p value**
Death, n (%)	157 (81.7)	83 (81.3)	74 (82.2)	0.87
Complete recovery of RF, n (%)	9 (25.7)	4 (21)	5 (31.2)	0.7
Partial recovery of RN, n (%)	24 (68.5)	13 (68.4)	11 (68.7)	0.7
Non-recovery of RF, n (%)	2 (5.7)	2 (10.5)	0	0.48

Values are presented in proportions.

AKI: acute renal injury, PHD: prolonged hemodialysis, and RF: renal function.

**Table 4:** Metabolic and volume control of the studied groups in the first three sessions of PHD.

	**G1 = 6 h (n = sessions)**			**G2 = 10 h (n = sessions)**			
	**S1** **(n = 104)**	**S2** **(n = 70)**	**S3** **(n = 38)**	**S1** **(n = 90)**	**S2** **(n = 63)**	**S3** **(n = 44)**	**p valu** **e** ^**∗**^
Predialysis Ur (mg/dl)	158 ± 59	123 ± 48	119 ± 44	137 ± 6^a^	102 ± 140^b^	89 ± 43^c^	NS
URR	0.6 ± 0.1	0.6 ± 0.1	0.56 ± 0.1	0.68 ± 0.1^d^	0.58 ± 0.1^b^	0.62 ± 0.1^f^	<.0001
Kt/V	1.05 ± 0.05	1.06 ± 0.05	1.03 ± 0.05	1.23 ± 0.06^d^	1.04 ± 0.05	1.18 ± 0.06^f^	0,01
Cr (mg/dl)	3.7 ± 1.4	2.8 ± 1.2	2.9 ± 1.1	3.6 ± 1.7^a^	2.5 ± 1^b^	2.4 ± 1.2^c^	NS
K (mEq/L)	4.7 ± 1	4.4 ± 0.9	4.3 ± 0.8	4.8 ± 1^a^	4.3 ± 0.8^b^	3.9 ± 0.6^f^	0.04
P (mEq/L)	6.5 ± 2.6	5.5 ± 2.1	5.8 ± 2.2	6 ± 2.5^a^	5.5 ± 3.2^b^	4.1 ± 1.6^f^	0.009
Bic (mEq/L)	18.3 ± 4.3	20.3 ± 4	20.3 ± 4	19.2 ± 4.3^a^	20.9 ± 5.5^b^	22.1 ± 3.5^c^	NS
pH	7.2 ± 0.1	8.4 ± 8.8	7.2 ± 0.09	7.2 ± 0.1^a^	7.2 ± 0.1^b^	7.2 ± 0.1^f^	0.03
Presc. UF (ml)	2064 ± 927	2262 ± 852	2217 ± 755	2580 ± 1000^d^	2626 ± 1123^e^	2656 ± 1004^f^	0.0002
Real UF (ml)	1791 ± 963	2032 ± 930	1940 ± 931	2345 ± 1017^d^	2097 ± 1494^b^	2240 ± 1295^c^	0.0006
Real UF (ml/h)	298 ± 160	338 ± 156	331 ± 149	234 ± 98	209 ± 147	224 ± 129	0.003
FB (1)	3.27 ± 1.9	-	1.33 ± 2.6	3.47 ± 1.7	-	0.47 ± 2.4	NS

Values are presented in mean and standard deviation.

PHD: prolonged hemodialysis, Ur: urea, URR: urea reduction ratio, Cr: creatinine, K: potassium, P: phosphorus, Bic: bicarbonate, UF: ultrafiltration, and Presc.: prescribed.

a: similar to S1 of G1, b: similar to S2 of G1, and c: similar to S3 of G1.

d: different from S1 of G1, e: different from S2 of G1, and f: different from S3 of G1.

NS: not significant (p >;0.05).

**Table 5:** Univariate logistic regression of clinical and laboratory characteristics, and dialysis complications associated with the death of patients with AKI treated with PHD.

**Parameter**	**OR**	**Confidence Interval**	**p value**
Age	1.01	0.99 – 1.03	0.24
Gender	0.60	0.28 – 1.28	0.19
Weight	1.01	1.00 – 1.03	0.04
Infectious focus	0.95	0.33 – 2.70	0.87
SAH	0.84	0.40 – 1.75	0.64
DM	0.73	0.31 – 1.74	0.48
CKD	0.22	0.02 – 1.76	0.15
ATN-ISS	0.94	0.11- 7.98	0.95
SOFA	1.22	1.06 – 1.40	0.004
Pre FB	1.47	1.12 – 1.93	0.004
Post FB	1.38	1.11 – 1.72	0.003
Pre Ur	0.99	0.99 – 1.0	0.15
Post Ur	1.00	0.99 – 1.01	0.67
Pre Cr	1.01	0.80 – 1.28	0.88
CR post	0.94	0.60 – 1.47	0.78
Pre K	1.79	1.18 – 2.73	0.006
Post K	1.82	0.82 – 4.00	0.13
Pre Bic	0.93	0.85 – 1.01	0.11
Post Bic	0.90	0.75 – 1.08	0.27
Pre UF	0.93	0.78 – 1.12	0.49
Post UF	0.86	0.67 – 1.12	0.28
RF outcome	1.0	<0,001 - >999,999	0.85
Hypotension	0.50	0.23 – 1.09	0.008
Coagulation	0.76	0.34 – 1.70	0.5
Hypokalemia	3.7	1.62 – 8.83	0.002
Hypophosphataemia	1.48	0.66 - 3.31	0.33

Or: odds ratio.

AKI: acute renal injury, PHD: prolonged hemodialysis, SAH: systemic arterial hypertension, DM: diabetes mellitus, CKD: chronic kidney disease, ATN-ISS: acute tubular necrosis individual severity score, SOFA: sequential organ failure assessment score, FB: fluid balance, Ur: urea, Cr: creatinine, K: potassium, Bic: bicarbonate, UF: ultrafiltration, and RF: renal function. Pre = 1st session, post = 3rd session of PHD.

**Table 6:** Multivariate logistic regression of clinical and laboratory characteristics and dialysis complications associated with the death of patients with AKI treated with PHD.

**Parameter**	**OR**	**Confidence Interval**	**p value**
Weight	1.01	0.98 – 1.04	0.51
SOFA	1.08	0.34 – 1.38	0.52
Pre FB	1.60	1.04 – 2.47	0.03
Post FB	1.54	1.16 – 2.04	0.002
Hypokalemia	1.69	0.39 – 7.22	0.47

Or: odds ratio.

AKI: acute kidney injury, HDP: prolonged hemodialysis, SOFA: sequential organ failure assessment score, and FB: fluid balance.

**Table 7:** Univariate logistic regression of clinical and laboratory characteristics and dialysis complications associated with the recovery of renal function in patients with AKI treated with PHD.

**Parameter**	**OR**	**Confidence Interval**	**p value**
Age	1.01	0.99 – 1.03	0.17
Gender	0.74	0.37 – 1.50	0.41
Weight	0.99	0.97 – 1.00	0.27
Infectious focus			
SAH	0.66	0.34 – 1.27	0.21
DM	0.98	0.48 – 1.97	0.95
CKD	1.9	0.52 – 7.2	0.32
ATN-ISS	1.17	0.15 – 9.18	0.87
SOFA	0.92	0.81 – 1.04	0.19
Pre FB	0.93	0.78 -1.10	0.41
Post FB	0.83	0.69 – 0.99	0.04
Pre Ur	1.00	0.99 – 1.00	0.71
Post Ur	0.99	0.98 – 1.00	0.65
Pre Cr	0.88	0.57 – 0.96	0.03
Post Cr	1.00	0.97- 1.03	0.57
Pre K	0.77	0.56 – 1.05	0.10
Post K	0.68	0.34 – 1.38	0,29
Pre Bic	1.07	0.99 – 1.15	0.08
Post Bic	1.00	0.85 – 1.18	0.92
Pre UF	1.09	0.94 – 1.27	0,24
Post UF	0.94	0.72 – 1.23	0,67
Hypotension	1.8	0.92 – 3.67	0,08
Coagulation	0.86	0.44 – 1.69	0.67
Hypokalemia	0.74	0.33 – 1.67	0.47
Hypophosphataemia	1.10	0.53 – 2.29	0.78

OR: odds ratio.

AKI: acute renal injury, PHD: prolonged hemodialysis, SAH: systemic arterial hypertension, DM: diabetes mellitus, CKD: chronic kidney disease, ATN-ISS: acute tubular necrosis individual severity score, SOFA: sequential organ failure assessment score, FB: fluid balance, Ur: urea, Cr: creatinine, K: potassium, Bic: bicarbonate, UF: ultrafiltration, and RF: renal function. Pre = 1st session, post = 3rd session of PHD.

**Table 8:** Multivariate logistic regression of clinical and laboratory characteristics and dialysis complications associated with the recovery of renal function in patients with AKI treated with PHD.

**Parameter**	**OR**	**Confidence Interval**	***p value***
SOFA	0.86	0.68-1.12	0.48
Pre FB	0.94	0.72-1.08	0.08
Post FB	0.98	0.65-0.97	0.009
Pre Cr	0.82	0.59-0.91	0.04

OR: odds ratio.

AKI: acute kidney injury, HDP: prolonged hemodialysis, SOFA: sequential organ failure assessment score, FB: fluid balance, and Pre Cr: creatinine at 1st PHD session.

## Data Availability

The data are available in UNESP thesis repository: http://hdl.handle.net/11449/151845.
